# Systemic low-grade C-reactive protein is associated with proximal symptom spread in carpal tunnel syndrome

**DOI:** 10.1097/PR9.0000000000001156

**Published:** 2024-04-10

**Authors:** Karolina Zvonickova, Amber Rhee, Oliver Sandy-Hindmarch, Dominic Furniss, Akira Wiberg, Annina B. Schmid

**Affiliations:** a Nuffield Department of Clinical Neurosciences, John Radcliffe Hospital, University of Oxford, Oxford, United Kingdom; b Department of Epidemiology, Johns Hopkins Bloomberg School of Public Health, Baltimore, MD, USA; c Nuffield Department of Orthopaedics, Rheumatology, and Musculoskeletal Sciences, University of Oxford, Oxford, United Kingdom

**Keywords:** Carpal tunnel syndrome, Neuropathic pain, Extraterritorial pain, Proximal pain, Inflammation

## Abstract

**Introduction:**

Neuropathic pain is a highly prevalent condition associated with persistent disability. Some patients with neuropathic pain experience symptom spread outside neuroanatomical boundaries; these patients report more severe sensory symptoms and greater disability. However, the mechanisms behind such symptom spread are not fully understood.

**Objective:**

We used pre-surgical carpal tunnel syndrome (CTS) as a human model system of neuropathic pain to identify differences in the concentration of serologic inflammatory mediators between patients with CTS with territorial symptoms and those with proximal symptom spread to either the elbow or shoulder/neck.

**Methods:**

We performed a post-hoc analysis, comparing levels of serologic inflammatory mediators in a discovery cohort among 3 symptoms spread profiles (n = 55; n = 25 no spread, n = 21 spread to elbow, n = 9 spread to shoulder/neck). We then de-novo analysed the significantly dysregulated mediators in an independent validation cohort (n = 72; n = 34 no spread, n = 16 spread to elbow, n = 22 spread to shoulder/neck).

**Results:**

The discovery cohort revealed higher serum concentrations of C-reactive protein (CRP) and interleukin-6 in patients with any symptom spread proximal to the wrist; interferon-γ was higher in patients with symptom spread to the elbow compared with those without proximal spread. The validation study replicated the association of higher CRP concentrations in patients with proximal spread to the elbow (no spread: median [interquartile range] 2.5 [5.4]; spread to elbow 6.2 [4.6]; spread to shoulder/neck 2.6 [3.7], *P* = 0.006). No other markers replicated in the validation cohort.

**Conclusions:**

Our findings suggest that proximal symptom spread in the context of neuropathic symptoms is associated with low-grade inflammation.

## Abstract 2

Supplemental Digital Content is Available in the Text.

C-reactive protein serum levels are higher in patients with carpal tunnel syndrome and proximal symptom spread. This suggests an association of extra-territorial symptoms with low-grade inflammation.

## 1. Introduction

Neuropathic symptoms caused by focal nerve injuries are usually perceived within the innervation territory of the affected nerve. However, a substantial proportion of patients experience extraterritorial spread outside the affected nerve territory^27,45,46^; up to 70% of patients with carpal tunnel syndrome (CTS) experience symptoms in a glove distribution^7^ and one-third of patients with lumbar and cervical radicular pain experience extradermatomal symptoms.^26^ Of interest, spread of symptoms proximal to the site of nerve injury is also highly prevalent (eg, up to 45% with CTS).^47^ Extraterritorial symptom spread is associated with more severe sensory symptoms and greater disability,^46^ yet the mechanisms behind such symptom radiation are not fully understood.

Among many possible mechanisms (eg, referred pain from somatic^16^ or vascular structures^23^), immune-mediated sensitisation of sensory afferents could contribute to the phenomenon of proximal symptom spread. Preclinical studies have provided increasing evidence supporting the role of the immune system in the initiation and maintenance of neuropathic pain.^9,11,24^ Neuroinflammation is active not only at the site of nerve injury but also at remote sites such as in the dorsal root ganglia,^13,14,34^ spinal cord,^13^ and higher pain centres.^10,25^ Such remote neuroinflammation may increase the excitability of intact neurons that originate from sites distant to the actual injury. Together with mechanisms of central sensitisation,^43^ hyperexcitability of intact neurons may explain the clinically observed extraterritorial symptom spread. However, there is still a paucity of evidence to support the role of remote neuroinflammation in the generation of extraterritorial symptoms in humans. Data from combined PET/MRI experiments identified remote neuroinflammatory changes at the level of the spinal cord or higher pain centres in humans with peripheral nerve injury.^1,2^ Early data suggest a potential link between cortical neuroinflammation and “fibromyalgianess,” which was largely defined by symptom severity and spread of pain.^2^ Further research is required to corroborate a link between neuroinflammation and symptom spread in patients with peripheral nerve injuries.

Carpal tunnel syndrome is an ideal human model system for studying associations between extraterritorial symptoms and inflammation. Although CTS symptoms arise from injury to the median nerve, sensations associated with CTS can be experienced both locally in the hand or extend proximally into the forearm or shoulder/neck.^47^ Such proximal spread, although common, cannot easily be explained by local median nerve mechanisms at the carpal tunnel.

The aim of this study was to identify differences in the concentration of serologic inflammatory mediators between patients with CTS with territorial symptoms and those with proximal symptom spread to either elbow or shoulder/neck. We conducted an exploratory analysis of protein expression of 20 systemic blood inflammatory markers in a discovery cohort. We subsequently validated the identified markers in an independent cohort. We identified C-reactive protein (CRP) as a main marker that is consistently upregulated in patients with proximal symptom spread to the elbow compared with those with distal symptoms. The discovery of variation in the immunological profiles of patients that differ in the localisation of symptoms could aid us in improving our understanding of proximal symptom spread and might have implications for treatment stratification.

## 2. Materials and methods

### 2.1. Participants

This cross-sectional study was performed in 2 existing CTS cohorts. The deeply phenotyped Oxford CTS Cohort^4,33^ served as the discovery cohort (London Riverside Ethics Committee, 10/H0706/35). The Oxford Molecular Genetics of CTS cohort^30^ (MGCTS, London—Camden & Kings Cross Research Ethics Committee, 16/LO/1920) served as the validation cohort. All participants gave informed written consent before participating.

### 2.2. Discovery cohort

Participants in the discovery cohort were recruited from waiting lists for carpal tunnel decompression surgery at Oxford University Hospitals NHS Foundation Trust. Patients were required to be 18 years or older, with clinically and electrodiagnostically confirmed CTS. Participants had to be willing and able to give informed consent. Participants with either insufficient command of English or insufficient mental capacity to obtain consent from or complete study questionnaires were not invited to participate. Participants were further excluded if they had any of the following: (1) electrodiagnostically confirmed nerve dysfunction other than CTS, (2) diagnosis of another condition affecting the upper limb/neck (eg, hand osteoarthritis, cervical radiculopathy; determined by a careful clinical examination), (3) self-reported diagnosis of autoimmune or inflammatory conditions (eg, rheumatoid arthritis, multiple sclerosis), (4) active infection (eg, hepatitis) and reported systemic infection (eg, flu) within 2 weeks before data collection, (5) diagnosis of systemic disease (eg, cancer, diabetes), (6) immunosuppressive medication prescription, (7) history of significant trauma to the upper limb or neck, (8) history of previous CTS surgery, or (9) if they were pregnant. Our final discovery sample included 55 patients with CTS after excluding patients with confounding comorbidities (n = 18).

### 2.3. Validation cohort

Similar to the discovery cohort, patients in the validation cohort were recruited from waiting lists for carpal tunnel decompression surgery at the Oxford University Hospitals NHS Foundation Trust. Inclusion and exclusion criteria for the cohort were consistent with those of the discovery cohort, apart from exclusion of systemic infection, which was not recorded. Also, no detailed clinical examination was recorded, and other concomitant diagnoses were excluded by self-report only. As per hand surgical practice, a clinical diagnosis of CTS was sufficient for inclusion, and electrodiagnostic tests were not routinely performed in the validation cohort. Only those with complete questionnaires, serum data and covariates (sex, body mass index [BMI], age) were included. Our final validation sample consisted of 72 patients with CTS after excluding participants with missing questionnaire or serum data (n = 25), participants with systemic diseases (n = 5), participants with other inflammatory conditions (n = 16), and participants with missing co-variates (n = 2). Flowcharts of both study populations can be found in Supplemental Figure 1, http://links.lww.com/PR9/A230.

### 2.4. Phenotypic data

The following phenotypic data were available from all participants: age, sex, height, weight, and BMI. The symptom subscale of the Boston Carpal Tunnel Syndrome Questionnaire (BCTQ) (0 = no symptoms to 5 = very severe symptoms) was used as an indicator of symptom severity.^19^ Neuropathic pain severity was evaluated with the Neuropathic Pain Symptom Inventory (NPSI; 0 = no pain to 10 = worst pain imaginable) for burning pain, deep pressure pain, paraesthesia, paroxysmal pain, evoked pain, and a composite score (0 = no pain to 100 = worst pain imaginable).^6^ Emotional well-being was examined with the Depression Anxiety and Positive Outlook Scale^31^ and the 13-item pain catastrophizing scale (PCS).^38^

In the discovery cohort, standard electrodiagnostic testing (EDT) of the median, ulnar, and radial nerve was performed with an ADVANCE system (Neurometrix, Woburn, MA). Electrodiagnostic test severity was graded on the scale derived by Bland et al.^5^ (grade 0 = normal, grade 6 = extremely severe). A more detailed description of the electrodiagnostic testing scale has been published previously.^33^

### 2.5. Classification of symptom spread

In both cohorts, the extent of symptom spread was assessed with a hand and body diagram (Supplementary Figure 2, http://links.lww.com/PR9/A230).^18^ While the patients completed these independently in the validation cohort, the participants in the discovery cohort completed these in the presence of a clinician, who prompted them to carefully consider any symptoms even if outside the affected hand. According to their body diagram, patients were classified as having either no proximal symptoms (symptoms limited distal to the wrist) or proximal spread to elbow or shoulder/neck.

### 2.6. Blood sampling and processing

Whole blood was collected through antecubital fossa venepuncture into gold top serum tubes (BD Vacutainer SST tube, Wokingham, United Kingdom) and allowed to clot for 15 to 30 minutes at room temperature. The tube was centrifuged for 10 minutes at 4°C for serum extraction. The serum fraction was aliquoted and stored at −80°C for batch processing.

In the discovery cohort, we performed an exploratory analysis on serum levels of 20 selected inflammatory markers available from a previous study^32^: interleukin-1β (IL-1β), IL-2, IL-4, IL-6, IL-8, IL-9, IL-10, IL-12, IL-17, CCL2 (C-C motif chemokine ligand 2), CCL5 (RANTES), C-X-C motif chemokine ligand 5 (CXCL5), CXCL10, fractalkine, granulocyte-macrophage colony stimulating factor (GM-CSF), vascular endothelial growth factor (VEGF), tumour necrosis factor (TNF), interferon-gamma (IFN-γ), transforming growth factor-beta (TGF-β), and CRP. These markers were selected for their associations with neuroinflammation in the current literature.

The methods for cytokine analysis have been described in detail previously.^32^ In short, U-PLEX plate custom biomarker multiplex assay kits (Meso Scale Diagnostics LLC, Rockville, MD) were used per standard protocol to detect 18 inflammatory mediators (IL1β, IL-2, IL-4, IL-6, IL-8, IL-9, IL-10, IL-12, IL-17, CCL2, CXCL5, CXCL10, fractalkine, GM-CSF, VEGF, TNF, and IFN-γ). Transforming growth factor-beta was run on a separate U-PLEX assay kit because it required an acidification step. The plates were read on a MESO QuickPlex SQ 120 plate reader (Meso Scale Diagnostics LLC) as per the manufacturer's instructions. The detection of CCL5 was performed with R-PLEX plates (Meso Scale Diagnostics LLC) following the standard manufacturer protocol. C-reactive protein was measured with a CRP Quantikine ELISA kit (R&D Systems, Minneapolis, MN) as per the standard protocol. The plates were read on a BMG FLUOstar Omega (BMG Labtech Ltd, Aylesbury, United Kingdom) with the wavelength set to 450 nm.

In the validation cohort, we de novo analysed the a priori selected 3 inflammatory markers—CRP, IL-6, and IFN-γ—from the discovery cohort that revealed statistically significant associations with proximal symptom spread. IL-6 was measured on a custom-designed U-PLEX custom biomarker multiplex assay kit (Meso Scale Diagnostics LLC). Interferon-gamma was run on a separate U-PLEX plate, and CRP was measured with the standard protocol for the CRP Quantikine ELISA kit (R&D Systems) as detailed before.^32^

All samples, including patient serum samples and standards in both the discovery and validation cohort, were run in duplicates. The standards contained known concentrations of each marker and were used to generate an 8-point standard curve. Although concentrations of the U-Plex plates were automatically calculated by the discovery workbench software for MSD plates, CRP levels were quantified using an interpolated calibration curve in GraphPad Prism (GraphPad Software Inc, version 9, La Jolla, CA).

### 2.7. Statistical analysis

Because this was a post hoc analysis of available cohorts, no sample size calculation was performed. All data were analysed using SPSS software (IBM, version 29, Chicago, IL). As only very few data points were missing, these were excluded from analysis with missingness indicated in tables. We conducted 2 separate Quade nonparametric ANCOVAs for the discovery and validation cohorts to compare serum levels of inflammatory mediators among patients without proximal symptom spread (symptoms limited distal to wrist), proximal symptom spread to the elbow, and proximal symptom spread to the shoulder/neck. Models were adjusted for sex, age (continuous), and BMI (continuous) to address the potential confounding effects of these covariates. As groups were comparable for anxiety, depression, and pain-related worrying, these were not adjusted for.

For the discovery cohort, a false discovery rate (FDR) correction for multiple testing was applied using the Benjamini–Hochberg correction, with FDR set to 0.25. For the validation cohort (only 3 inflammatory markers), Bonferroni corrections were applied. Least significant difference (LSD) post hoc tests were used to identify significant group differences.

## 3. Results

Clinical data for the 55 patients with CTS from the discovery cohort and 72 patients from the validation cohort were comparable for age, sex, and BMI (supplemental Table 1, http://links.lww.com/PR9/A230).

In the discovery cohort, 25 patients (45%) were grouped as having territorial symptoms only, 21 (38%) as having proximal symptom spread to the elbow, and 9 (16%) as having proximal symptom spread to the shoulder/neck (Table 1). In the validation cohort, 34 patients (47%) were grouped as having no proximal symptom spread, 16 (22%) as having proximal symptom spread to the elbow and 22 (31%) as having proximal symptom spread to the shoulder/neck.

**Table 1:** Baseline clinical characteristics of cohorts by pain spread group, presented as median with interquartile range unless indicated otherwise.

	Discovery cohort			Validation cohort		
	No proximal spread (N = 25)	Proximal spread to elbow (N = 21)	Proximal spread to neck (N = 9)	No proximal spread (N = 34)	Proximal spread to elbow (N = 16)	Proximal spread to neck (N = 22)
Sex						
Male (%)	12 (48.0)	3 (14.3)	3 (33.3)	13 (38.2)	7 (43.8)	5 (22.7)
Female (%)	13 (52.0)	18 (85.7)	6 (66.7)	21 (61.8)	9 (56.3)	17 (77.3)
Age (y)	67.0 [14.0]	64.0 [17.0]	60.0 [12.0]	60.5 [28.8]	56.0 [29.8]	52.0 [22.0]
Mean BMI (SD) (kg/m^2^)	25.5 (4.4)	25.7 (5.8)	26.0 (4.8)	28.4 (5.6)	30.1 (8.1)	27.2 (4.6)
Symptom duration (y)	3.0 [3.3]	3.5 [3.5]	3.0 [3.5]	NA	NA	NA
Boston symptom score	2.3 [0.7]	2.6 [0.9]	3.3 [0.5]	2.9 [0.9]	3.5 [0.8]	3.2 [0.7]*
Boston function score	1.9 [0.9]	2.1 [1.0]	2.8 [1.8]	2.2 [1.2]	3.1 [0.5]	2.4 [1.0]*
EDT grade	4.0 [2.0]	3.0 [2.0]	3.0 [1.0]	NA	NA	NA
NPSI score						
Total score	8.0 [6.8]	12.8 [13.2]	17.3 [9.5]	13.0 [12.3]	19.7 [12.9]	12.0 [10.7]‡
Burning pain	0.0 [2.0]	0.0 [3.0]	3.0 [3.0]	3.0 [4.0]	0.5 [6.5]	0.0 [5.0]*
Deep pressure pain	0.0 [1.5]	2.5 [4.0]	2.0 [5.0]	1.5 [4.5]	4.5 [6.5]	2.0 [4.1]†
Evoked pain	0.0 [1.7]	1.3 [3.0]	1.7 [2.7]	2.0 [4.3]	2.2 [5.3]	2.5 [5.3]†
Paraesthesia	5.0 [4.5]	6.0 [5.5]	7.0 [4.0]	6.8 [4.8]	7.5 [2.3]	7.8 [4.1]†
Paroxysmal pain	0.0 [1.0]	0.5 [4.0]	2.0 [2.5]	1.3 [4.0]	3.0 [4.8]	1.5 [3.5]*
DAPOS score						
Depression	5.0 [1.0]	7.0 [3.0]	6.0 [3.0]	5.0 [2.0]	5.0 [7.3]	5.5 [4.0]
Anxiety	3.0 [0.0]	3.0 [1.0]	3.0 [1.0]	3.0 [2.0]	3.0 [3.8]	3.0 [3.8]
Positive outlook	13.0 [2.0]	12.0 [4.0]	12.0 [2.0]	11.0 [5.0]	11.0 [4.3]	12.0 [2.8]
Pain catastrophizing scale	3.0 [14.0]	9.0 [19.0]	9.0 [4.0]	6.0 [23.5]	13.0 [24.5]	10.0 [14.5]

* Missing data on 1 patient.

† Missing data on 2 patients.

‡ Missing data on 3 patients.

DAPOS, depression, anxiety, and positive outlook scale; EDT, electrodiagnostic testing; IQR, interquartile range; NA: not applicable; NPSI, neuropathic pain symptom inventory; PCS, pain catastrophizing scale.

### 3.1. Serologic changes between people with no spread and different extents of proximal symptom spread in the discovery cohort

**Figure 1.**
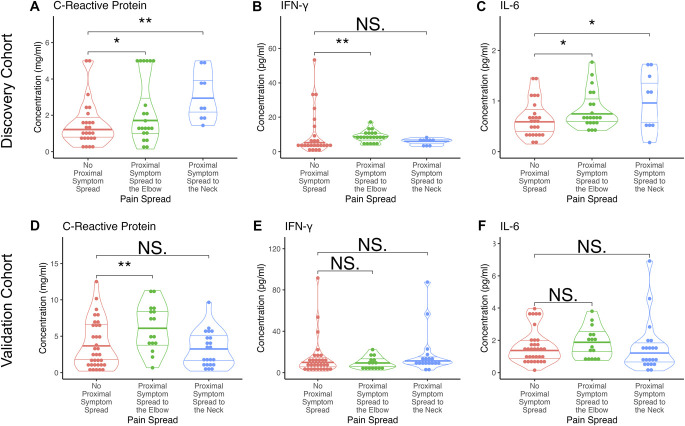
Concentrations of statistically significant different serum inflammatory mediators between people with CTS without and with proximal symptom spread to the elbow or shoulder/neck in the discovery and validation cohorts. (A–C) Violin graphs (median and quartiles) of serum concentrations of CRP, IFN-γ, and IL-6 in the discovery cohort according to symptom spread group. (D–F) Serum concentrations of these same markers in the validation cohort by symptom spread group. *P value <0.05, **P value <0.01 in LSD post hoc tests. CTS, carpal tunnel syndrome; CRP, C-reactive protein; IFN-γ, interferon-γ; IL-6, interleukin-6; NS, non significant.

Table 2 contains the results of the serum protein expression analyses comparing CTS patients with no symptom spread with patients with proximal symptom spread to the elbow or shoulder/neck. The serum concentrations of 3 inflammatory mediators were significantly different in patients with pain spread when compared with patients without pain spread: IL-6 (*P* = 0.03), IFN-γ (*P* = 0.017), and CRP (*P* = 0.002, Fig. 1A–C). Among these mediators, IL-6, IFN-γ, and CRP were elevated in patients with CTS with symptom spread to the elbow when compared with patients with no symptom spread (Fig. 1A–C). IL-6 and CRP serum concentrations were also higher in patients with CTS with symptom spread to the shoulder and/or neck compared with those without proximal pain spread (Fig. 1A, C).

**Table 2:** Serum level inflammatory mediators among groups without proximal symptom spread and different extents of proximal symptom spread in the discovery cohort.

Assay	Median concentration* [IQR]			*P*		
	No proximal symptom spread (N = 25)	Proximal symptom spread to elbow (N = 21)	Proximal symptom spread to neck (N = 9)	Quade nonparametric ANCOVA	No spread vs elbow LSD Post hoc	No spread vs shoulder/Neck LSD post hoc
**CRP**	**1.1 [0.9]**	**1.3 [3.9]**	**2.4 [1.9]**	**0.002**	**0.028**	**0.001**
**IFN-γ**	**4.0 [6.2]**	**8.8 [2.6]**	**6.4 [2.9]**	**0.017**	**0.006**	0.887
**IL-6**	**0.6 [0.4]**	**0.7 [0.4]**	**1.1 [1.0]**	**0.03**	**0.022**	**0.038**
IL-8	8.0 [3.0]	10.3 [3.4]	11.9 [6.4]	0.08	0.084	0.051
TNF	0.4 [0.3]	0.6 [0.3]	0.5 [0.6]	0.222	0.114	0.225
IL-4	0.1 [0.1]	0.1 [0.1]	0.1 [0.0]	0.272	0.113	0.426
GM-CSF	0.0 [0.0]	0.0 [0.1]	0.0 [0.0]	0.279	0.847	0.156
IL-2	0.0 [0.0]	0.0 [0.0]	0.0 [0.0]	0.327	0.193	0.818
IL-10	0.1 [0.1]	0.2 [0.2]	0.1 [0.1]	0.33	0.158	0.951
IL-1β	0.1 [0.1]	0.1 [0.1]	0.1 [0.1]	0.377	0.281	0.229
IL-17	0.0 [0.0]	0.0 [0.0]	0.0 [0.2]	0.401	0.888	0.192
IL-12	0.0 [0.2]	0.2 [0.4]	0.0 [0.2]	0.545	0.284	0.878
CXCL5	1437.8 [997.5]	1356.1 [903.6]	1815.4 [737.2]	0.59	0.864	0.315
TGF-β	10857.3 [4406.7]	12577.1 [4010.4]	10525.7 [2737.7]	0.601	0.325	0.876
RANTES	2841.5 [2191.1]	2800.9 [1531.5]	1825.0 [1747.2]	0.603	0.867	0.385
Fractalkine	6079.9 [1751.3]	6574.9 [2347.0]	6392.8 [2144.4]	0.715	0.42	0.697
CCL2	288.9 [75.3]	317.4 [257.9]	264.2 [113.9]	0.724	0.426	0.723
IL-9	0.3 [0.4]	0.2 [0.2]	0.2 [0.4]	0.785	0.605	0.541
VEGF	97.3 [89.5]	90.9 [46.9]	92.8 [98.0]	0.887	0.835	0.628
CXCL10	268.0 [190.9]	233.0 [147.3]	233.8 [146.6]	0.912	0.675	0.947

Markers that were significantly different among groups after BH-correction (FDR = 0.25) are highlighted in bold (overall ANCOVA adjusted for sex, age, and BMI).

* Concentrations of markers are in pg/mL with the exception of CRP, which is in mg/L.

CCL2, C-C motif ligand 2; CRP, C-reactive protein; CXCL10, CXC motif chemokine 10; CXCL5, CXC motif chemokine 5; GM-CSF, granulocyte macrophage-colony stimulating factor; IFN-γ, interferon-γ; IL-10, interleukin-10; IL-12, interleukin-12; IL-17, interleukin-17; IL-1β, interleukin-1β; IL-2, interleukin-2; IL-4, interleukin-4; IL-6, interleukin-6; IL-8, interleukin-8; IL-9, interleukin-9; IQR, interquartile range; LSD, least significant difference; RANTES, regulated upon activation, normal T-cell expressed and presumably secreted (also known as CCL5); TGF-β, transforming growth factor-β; TNF, tumor necrosis factor; VEGF, vascular endothelial growth factor.

### 3.2. Serologic changes between people with no spread and different extents of proximal symptom spread in the validation cohort

The results of the validation cohort of the serum levels of the 3 identified cytokines are shown in Table 3. We validated a group difference of CRP (*P* = 0.006), with elevated CRP levels in patients with symptom spread to the elbow compared with patients with no proximal symptom spread (Fig. 1D). Unlike in the discovery cohort, the association between CRP serum levels and symptom spread to the shoulder/neck were not statistically significant. No other findings were validated.

**Table 3:** Serum level inflammatory mediators between people with proximal and no proximal symptom spread in the validation cohort.

Assay	Median concentration* [IQR]			*P*		
	No proximal symptom spread (N = 34)	Proximal symptom spread to elbow (N = 16)	Proximal symptom spread to neck (N = 22)	Quade nonparametric ANCOVA	No spread vs elbow LSD post hoc	No spread vs shoulder/neck LSD post hoc
CRP	2.5 [5.4]	6.2 [4.6]	2.6 [3.7]	0.006	0.006	0.570
IL-6	1.4† [1.1]	1.8 [1.4]	0.9† [1.0]	0.068	0.104	0.321
IFN-γ	8.9§ [9.2]	7.5† [6.6]	10.8‡ [5.9]	0.174	0.703	0.116

Markers that were significantly different among groups after Bonferroni correction (α = 0.017) are highlighted in grey (overall ANCOVA adjusted for sex, age, and BMI).

* Concentrations of markers are in pg/mL with exception of CRP, which is in mg/L.

† Missing data on 1 patient.

‡ Missing data on 4 patients.

§ Missing data on 5 patients.

CRP, C-reactive protein; IFN-γ, interferon-γ; IL-6, interleukin-6; IQR, interquartile range; LSD, least significant difference.

## 4. Discussion

Using CTS as a human model system, our discovery cohort identified raised levels of proinflammatory markers CRP, IFN-γ, and IL-6 in association with symptom spread. C-reactive protein and IL-6 were higher in patients with any symptom spread proximal to the wrist, whereas IFN-γ was only higher in patients with symptom spread to the elbow compared with those without proximal symptom spread. In an independent validation cohort, we confirmed a group difference for CRP, with heightened levels associated with proximal symptom spread to the elbow. The associations for the other cytokines were not replicated. This indicates that a systemic low-grade inflammatory response may be associated with proximal symptom spread to the elbow in the context of peripheral nerve injury and neuropathic pain.

It is well established that neuropathic pain is frequently associated with extraterritorial symptom spread in a range of conditions.^26,47^ In our CTS cohorts, 54% and 52% of patients in the discovery and validation cohort, respectively, reported symptoms that extended proximal to the wrist. This high prevalence of proximal symptoms in CTS is in line with previous reports (37%–63%).^8,21,27,28,36,45,47^ It has been argued that proximal symptoms may simply be attributed to the coexistence of other proximal conditions.^28^However, this theory is questioned because both distal and proximal symptoms in 94% of patients with proximal symptoms disappear after carpal tunnel decompression.^8^ Furthermore, the prevalence of proximal symptoms is similarly as high in cohorts where coexisting disorders are carefully excluded (∼37–45%).^45,47^ In our cohorts, particularly the discovery cohort, we were very careful to exclude potentially confounding coexisting disorders, making the mere coexistence of other conditions an unlikely explanation for our findings.

In the absence of coexisting conditions, several mechanisms can explain extraterritorial spread of symptoms, including somatic and visceral referred pain (eg, from vascular structures) and the presence of central sensitisation.^22^ Indeed, patients with symptom spread outside the median nerve distribution in the hand show widespread thermal and mechanical hyperalgesia and enhanced temporal summation^45^; however, this is not the case for patients with proximal spread. The preclinical literature suggests that neuroimmune changes at sites distant to the original nerve injury play a role in the context of peripheral neuropathic pain.^9,11,24^ This is supported by preliminary findings of elevated levels of first- and second-generation neuroinflammation translocator protein radioligands (TSPO) at the level of the spinal cord and somatosensory cortex in people with lumbar radicular leg pain.^1^ The authors identified a positive correlation between somatosensory cortical TSPO signal and “fibromyalgianess” as determined by the American College of Rheumatology Fibromyalgia Survey Criteria.^42^ In addition to symptom severity, these survey criteria heavily rely on the presence of widespread pain. The findings imply an association between central neuroinflammation and spread of symptoms in patients with lumbar radicular pain.

Our data of systemic inflammatory markers corroborates a role of low-grade inflammation in the context of symptom spread. In particular, CRP could be replicated in our independent validation cohort. Human CRP is one of the main human inflammatory reactants and serves as both a sensor and initiator of the innate immune response, rising in concentration during an inflammatory response.^35^ C-reactive protein is altered in numerous conditions (eg, cardiovascular, respiratory, gastrointestinal, autoimmune), including in entrapment neuropathies such as sciatica.^17,37^ We and other authors have previously not found it elevated in people with chronic CTS.^3,32^ Yet, our current data suggest that there is heterogeneity of CRP levels within the CTS population, even after adjustment for BMI, which is significantly associated with altered CRP levels.^41^ Experimental peripheral nerve injury suggests a causal link between CRP and neuropathic pain; CRP expression is rapidly and long-lastingly elevated within dorsal root ganglia following peripheral nerve injury, while knocking down CRP expression suppresses nerve injury-induced hyperalgesia.^20^ The methodology in this study cannot ascertain a causal link, yet it confirms that CRP levels differ among CTS patients with distinct symptom distributions.

### 4.1. Strengths and limitations

The independent replication of CRP in the validation cohort corroborates its association with proximal pain spread. There are several possible explanations for why the other dysregulated cytokines and the association of higher CRP with spread to the neck from the discovery cohort did not replicate. First, these could have been spurious findings generated by multiple testing in the relatively small discovery cohort. Second, although the discovery cohort was very carefully diagnosed and phenotyped, the validation cohort was more heterogeneous. For instance, the hand diagrams were self-completed without investigator guidance. Several hand diagrams in the validation cohort were limited to crosses over some areas rather than careful shading. This made it difficult to ascertain a connection of the proximal (and in particular, neck) symptoms with patients' hand symptoms and may have led to misclassification of some patients. Indeed, spread to the neck was more prevalent than spread to the elbow in the validation cohort, which diverges from the discovery cohort and previously published data.^28^ Also, patients in the validation cohort did not routinely undergo electrodiagnostic testing. Therefore, it is possible that territorial symptoms from undetected ulnar and/or radial nerve injury might have been misinterpreted as proximal symptom spread from median nerve injury. Furthermore, the presence of coexisting disorders was predominantly screened through patients' self-report. This may decrease confidence in the exclusion of coexisting conditions, which could have influenced the results. Nevertheless, the proportion of patients with proximal pain was comparable between the 2 cohorts, although some variation in its extent (to the elbow vs shoulder/neck) was apparent.

In the absence of access to neural tissue, serum inflammatory mediators serve as proxies for, but may not accurately reflect, their concentrations within the neuraxis. Some of the examined mediators do not circulate at high levels in the blood and, therefore, may not have been detectable by the MSD plate reader. Furthermore, our list of serologic inflammatory markers was not exhaustive, and some are highly variable even among healthy people.^44^ It is therefore notable that we identified and independently replicated CRP to be associated with proximal symptom spread.

For CRP analyses, we encountered a ceiling effect in the assays used in the discovery cohort. Therefore, we used a wider range assay in the validation cohort. Although this explains the slightly higher values in the validation cohort, this would not have influenced our within-cohort analyses, which were all done on the same assays and during the same experiment, thus providing consistent internal controls.

### 4.2. Future directions and potential clinical implications

Future work will have to determine whether symptom patterns may aid intervention stratification, a main ambition in the development of personalised pain management.^40^ Although current trial evidence suggests that nonsteroidal anti-inflammatory drugs (NSAIDs) are largely ineffective for people with CTS^12,29^ and oral steroids and steroid injections have short term benefits,^15^ group effects reveal large variation in effectiveness, potentially reflecting response heterogeneity. Furthermore, survey data suggest that, although NSAIDs are prescribed only to a minority of patients with CTS,^39^ most report improvement. Given our findings of low-grade inflammation in people with CTS and proximal symptom spread, future studies are warranted to examine whether symptom localisation can be used to stratify patients for these anti-inflammatory treatments. In addition, the role of other mechanisms potentially contributing to proximal symptom spread in focal nerve injuries such as somatic or visceral referred pain should be evaluated.

## 5. Conclusion

Using CTS as a model system and carefully validating our findings in an independent patient cohort, we identified elevated levels of CRP to be associated with proximal spread of symptoms to the elbow. These results suggest that low-grade systemic inflammation might play a role in extraterritorial symptom spread in people with peripheral nerve injuries and neuropathic pain. Future studies need to examine whether symptom localisation is a useful tool to stratify pain management.

## Disclosures

The authors have no conflict of interest to declare.

## Supplementary Material

SUPPLEMENTARY MATERIAL

## Supplemental digital content

Supplemental digital content associated with this article can be found online at http://links.lww.com/PR9/A230.
