# Supplementary material for: Systemic low-grade C-reactive protein is associated with proximal symptom spread in carpal tunnel syndrome
Source: Pain Rep. 2024 Apr 10;9(3):e1156. doi: 10.1097/PR9.0000000000001156 (PMC11008662; doi:10.1097/PR9.0000000000001156)
Supplement: SUPPLEMENTARY MATERIAL [file painreports-9-e1156-s001.pdf]

Supplementary Material

Supplemental Figure 1. Flowchart of the discovery and validation cohorts

(A) Discovery cohort

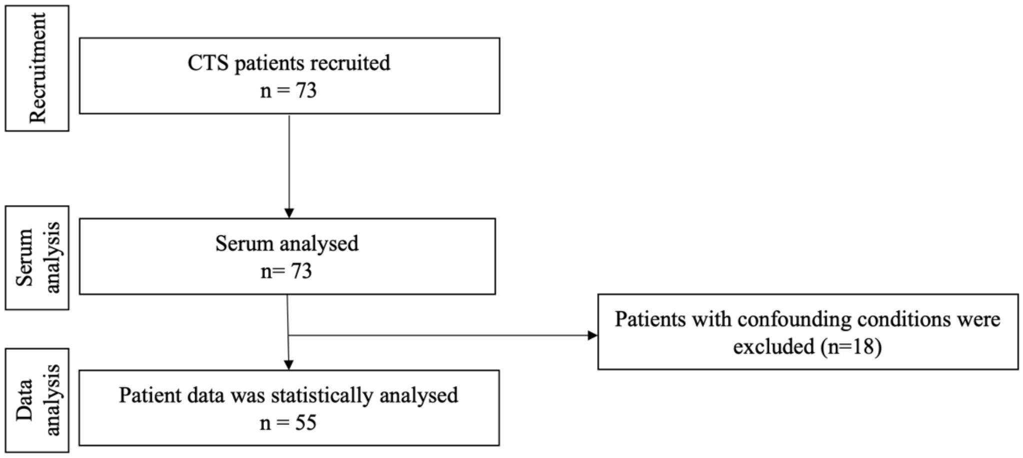

(B) Validation Cohort

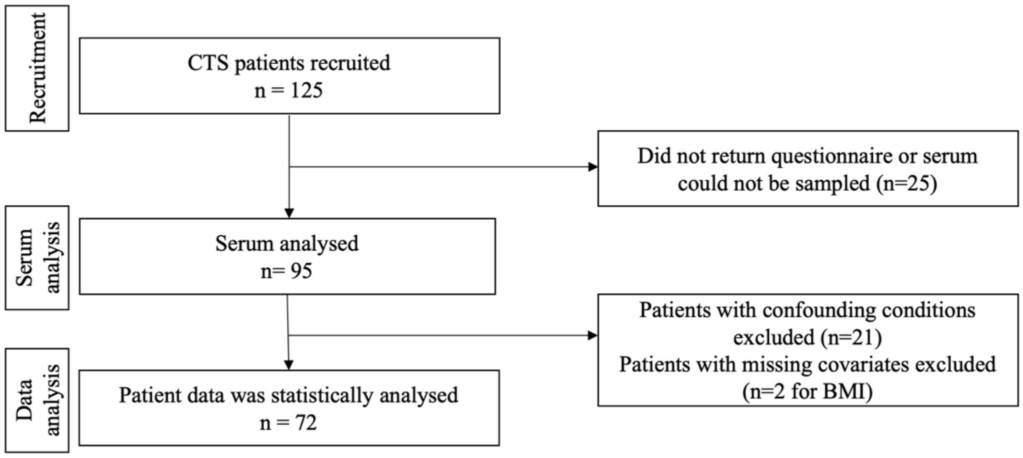

Supplementary Figure 2. Hand/body diagram

ID______________

Hand/body diagram

Please mark as accurately as possible the area of pain/tingling/numbness

Do you have predominant pain☐ or tingling☐ or numbness☐ which is your dominant hand: right☐ left☐ both☐

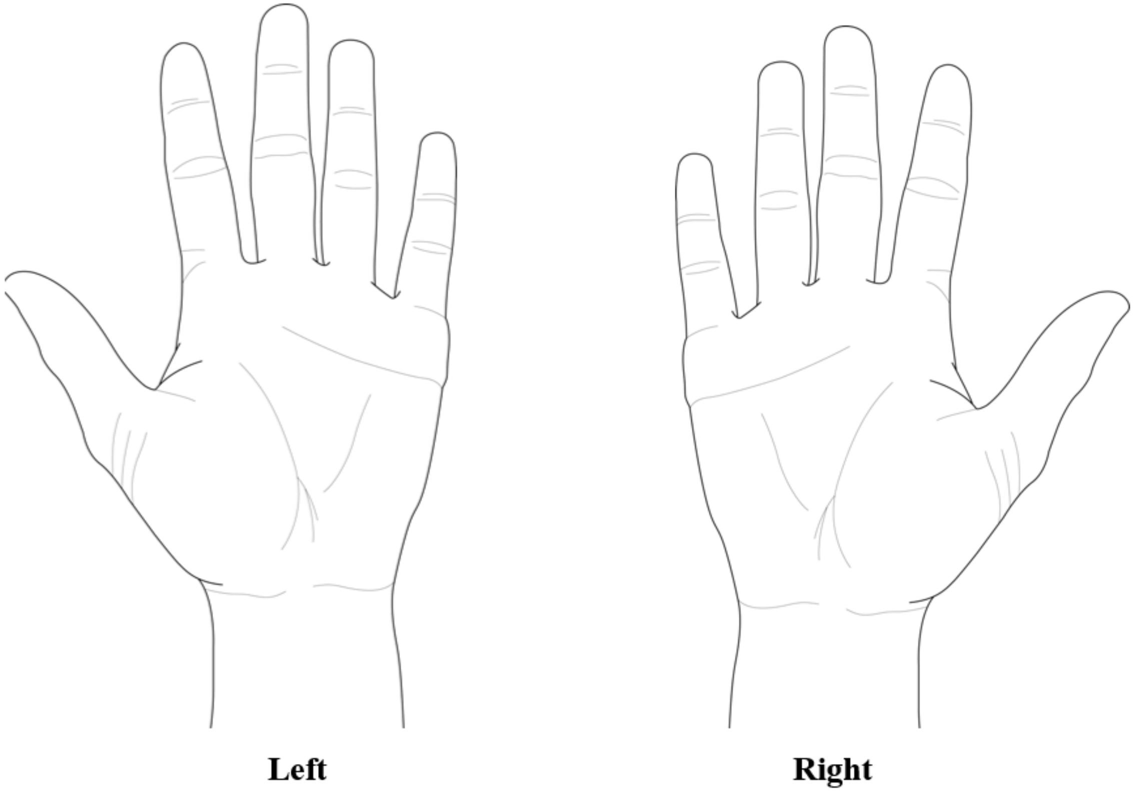

ID_____________

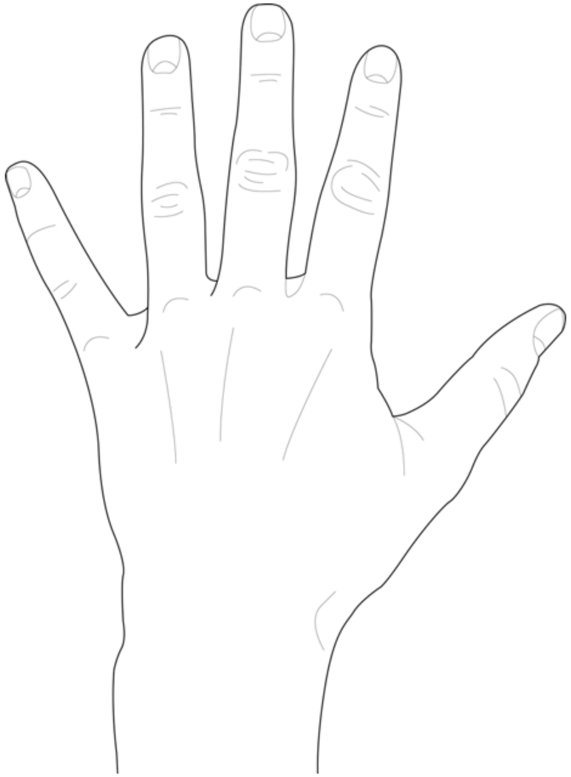

Left

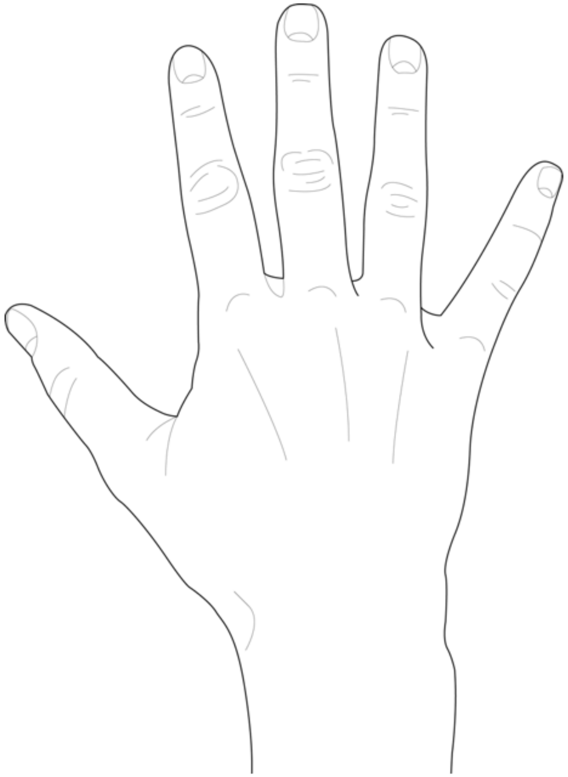

Right

ID_____________

Does your pain radiate to other regions of your body? Yes ☐ No ☐

Please mark all areas where you feel symptoms

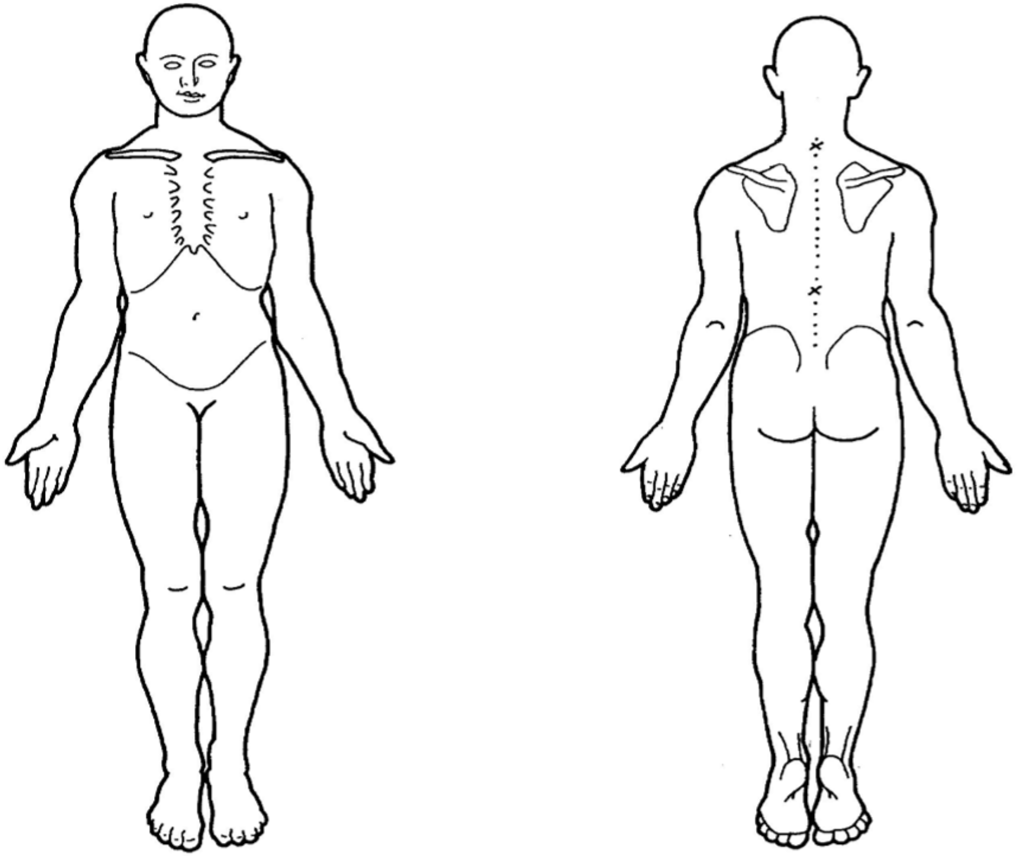

Supplemental Table 1: Clinical data of the discovery and validation cohorts, presented as median with interquartile range [IQR] unless indicated otherwise

	**Discovery (N=55)**	**Validation (N=72)**
**Sex**		
Male (%)	18 (32.7)	25 (34.7)
Female (%)	37 (67.3)	47 (65.3)
**Age (years)**	64.0 [15.0]	58.0 [28.5]
**Mean BMI (SD) (kg/m2)**	25.7 (5.0)	28.4 (6.0)
**Boston Symptom Score**	2.5 [1.1]	3.1 [1.0]
**Boston Function Score**	2.1 [1.2]	2.6 [1.3]
**EDT grade**	3.0 [2.0]	NA [NA]
**NPSI Score**		
Total Score	9.7 [12.4]	13.5 [12.8]
Burning Pain	0.0 [4.0]	1.0 [5.0]
Deep Pressure Pain	1.5 [2.8]	2.0 [5.0]
Evoked Pain	0.3 [2.7]	2.2 [4.8]
Paraesthesia	6.0 [4.8]	7.0 [4.0]
Paroxysmal Pain	0.0 [3.0]	1.5 [4.3]

IQR, interquartile range; SD, standard deviation; EDT, electrodiagnostic testing; VAS, visual analogue scale; NPSI, Neuropathic Pain Symptom Inventory
